# Humoral protection against mosquito bite-transmitted *Plasmodium falciparum* infection in humanized mice

**DOI:** 10.1038/s41541-017-0028-2

**Published:** 2017-10-09

**Authors:** Brandon K. Sack, Sebastian A. Mikolajczak, Matthew Fishbaugher, Ashley M. Vaughan, Erika L. Flannery, Thao Nguyen, Will Betz, Mary Jane Navarro, Lander Foquet, Ryan W. J. Steel, Zachary P. Billman, Sean C. Murphy, Stephen L. Hoffman, Sumana Chakravarty, B. Kim Lee Sim, Marije Behet, Isaie J. Reuling, Jona Walk, Anja Scholzen, Robert W. Sauerwein, Andrew S. Ishizuka, Barbara Flynn, Robert A. Seder, Stefan H. I. Kappe

**Affiliations:** 1 Center for Infectious Disease Research, Seattle, WA USA; 2 Departments of Laboratory Medicine and Microbiology and the Center for Emerging and Re-emerging Infectious Diseases, University of Washington, Seattle, WA USA; 3 Sanaria, Inc., Rockville, MD USA; 4 Radboud University, Nijmegen, The Netherlands; 5 National Institutes of Health, Bethesda, MD USA; 6 Department of Global Health, University of Washington, Seattle, WA USA

## Abstract

A malaria vaccine that prevents infection will be an important new tool in continued efforts of malaria elimination, and such vaccines are under intense development for the major human malaria parasite *Plasmodium falciparum* (*Pf*). Antibodies elicited by vaccines can block the initial phases of parasite infection when sporozoites are deposited into the skin by mosquito bite and then target the liver for further development. However, there are currently no standardized in vivo preclinical models that can measure the inhibitory activity of antibody specificities against *Pf* sporozoite infection via mosquito bite. Here, we use human liver-chimeric mice as a challenge model to assess prevention of natural *Pf* sporozoite infection by antibodies. We demonstrate that these mice are consistently infected with *Pf* by mosquito bite and that this challenge can be combined with passive transfer of either monoclonal antibodies or polyclonal human IgG from immune serum to measure antibody-mediated blocking of parasite infection using bioluminescent imaging. This methodology is useful to down-select functional antibodies and to investigate mechanisms or immune correlates of protection in clinical trials, thereby informing rational vaccine optimization.

## Malaria: ‘Humanized’ mice offer a new preclinical research tool

Mice containing human liver cells can model early-stage malaria infection and test antibody efficacy. A major obstacle in malaria vaccine development is the lack of relevant preclinical models to study how to prevent malaria infection. Stefan Kappe, of the United States’ Center for Infectious Disease Research and the University of Washington, led a collaboration of American and Dutch scientists to overcome this using mice in which the mouse liver cells have been largely replaced with human liver cells. The group demonstrated that their model mirrors infection with the malaria-causing parasite *Plasmodium falciparum* from mosquito bite through the week-long liver development, and harnessed this to discern the efficacy of different antibodies against the parasite. This research could hugely benefit our understanding of malaria infection and reduce the high failure rate of human vaccine clinical trials.

## Introduction

Despite considerable effort and substantial progress in reducing the malaria burden in many countries over the past decade, more than 200 million people still suffered from this parasitic disease in 2015, resulting in over 400,000 deaths due in large part to infection with *Plasmodium falciparum* (*Pf*) (WHO 2015). A vaccine capable of preventing malaria infection would prevent disease, death and onward parasite transmission and as such would constitute a critical tool in disease reduction as well as parasite eradication. However, all licensed vaccines to date have targeted viruses and bacteria, and although malaria vaccine development has seen some recent progress, a vaccine capable of delivering high levels of durable protection from *Plasmodium* parasites has yet to be developed.

During their complex life cycle within the mammalian host, malaria parasites present multiple targets for antibody-mediated interference with infection, providing a strong rationale that antibody-based vaccines could effectively interrupt the parasite transmission cycle and prevent disease and death. Whole attenuated parasite vaccine candidates and subunit vaccine candidates can both elicit protective antibody responses capable of neutralizing or destroying the parasite during infection.^1–8^ Attenuated parasites stimulate a broad antibody and T cell-mediated adaptive immune response against numerous parasite antigens. Subunit vaccines constitute a narrower approach where recombinant or vectored parasite antigen(s) are formulated with an immune-stimulatory adjuvant to elicit an antigen-specific response.

Antibodies can block *Plasmodium* parasite infection in the skin immediately after transmission, which occurs when an infected *Anopheles* mosquito injects tens to a few hundred sporozoite stages during a bite. Sporozoites are highly motile and traverse multiple cell types in search of a blood vessel, which gains them access to the blood circulation. In rodent models of malaria, it was shown that antibodies targeting the sporozoite can effectively prevent passage of the sporozoite to the liver by reducing the number of sporozoites ejected from the mosquito proboscis and also immobilizing the sporozoite in the dermis, thereby preventing their access to the circulation.^9,10^ Sporozoite motility and cell traversal are processes that require unique secreted and membrane-anchored proteins, which might be targeted with antibodies to prevent access to the blood circulation.^11,12^ Once in the circulation, sporozoites are rapidly transported to the liver where they again traverse multiple cell types as they cross the liver sinusoidal barrier and then infect a suitable hepatocyte. Leaving the circulation to enter the liver parenchyma also presents an opportunity for antibody-mediated prevention of infection as sporozoites are exposed to circulating antibodies that could target multiple sporozoite proteins involved in cell traversal and invasion, potentially preventing hepatocyte infection and in consequence, parasite replication in the liver. This in turn prevents the release of exo-erythrocytic merozoites and the establishment of a blood stage infection and its associated mortality and morbidity.^13^

The sporozoite and liver stages, collectively called the pre-erythrocytic (PE) stages of the parasite, are asymptomatic and are therefore attractive vaccine targets. Preventing the PE stages of infection will prevent all disease and the establishment of circulating sexual stage parasite populations that are transmissible. Indeed, endeavors are underway to identify new antibody targets for PE stages based on comprehensive sporozoite surface proteome and secretome data and a more in depth molecular understanding of the biological processes of parasite cell traversal and hepatocyte invasion.^14,15^ However, preclinical laboratory assays for assessing infection-inhibiting antibodies are extremely limited. Traditionally, rodent malaria models have been used for active immunization or for passive transfer of polyclonal or monoclonal antibodies followed by sporozoite challenge. However, there is significant evolutionary divergence between the malaria parasite species that infect rodents and humans, limiting the rodent malaria models as predictive preclinical experimental systems. Transgenic rodent malaria parasites carrying individual *Pf* antigens of interest have been created as bridging tools, but these are limited in the type and number of antigens that can be assessed.^16–18^ Thus, preclinical in vivo models that directly assess antibody efficacy against *Pf* challenge could be extremely helpful to inform which antigen candidates are to be advanced to clinical trials using controlled human malaria infection (CHMI).^19^ Without robust preclinical evidence for protective efficacy of subunit vaccine candidates against *Pf* challenge, CHMI trials have resulted in numerous vaccine candidate failures in the past.^20–25^ Although in vitro assays to evaluate antibody-mediated protection against *Pf* sporozoite infection have been established using monocultures of hepatoma cells or primary human hepatocytes^26^ they do not recapitulate the sporozoites infection route from the skin to the liver and therefore cannot capture antibody activities that block the parasite prior to hepatocyte infection.^13,27,28^

A bridge between preclinical in vitro assays and human CHMI trials could be built with liver-humanized mice, which fully support *Pf* PE infection.^29,30^ Measurement of parasite liver stage burden can then be used to assess passively transferred antibody efficacy.^6,31,32^ We have previously shown that these liver-chimeric humanized mice (referred to as FRG huHep mice), when challenged with a luciferase-expressing *Pf* parasite can be used to detect antibody-mediated reduction in liver infection by bioluminescent imaging.^2,29,33,34^ Here, we use FRG huHep mice to develop a robust platform to evaluate the protective efficacy of antibodies against natural *Pf* infection. We demonstrate that this model can differentiate monoclonal antibodies (mAbs) of varying infection-blocking activity and measure the infection-blocking activity of polyclonal immune IgG from human volunteers immunized with whole sporozoites. We also have adapted this model to measure antibody-mediated sterile protection using a physiologically relevant challenge dose. Importantly, mosquito bite challenge of FRG huHep mice discriminated antibody performance that was superior to in vitro assays, indicating the importance of using the natural *Pf* parasite infection route when assessing interventions against PE infection.

## Results

### *Pf* liver infection following mosquito bite challenge

**Fig. 1:**
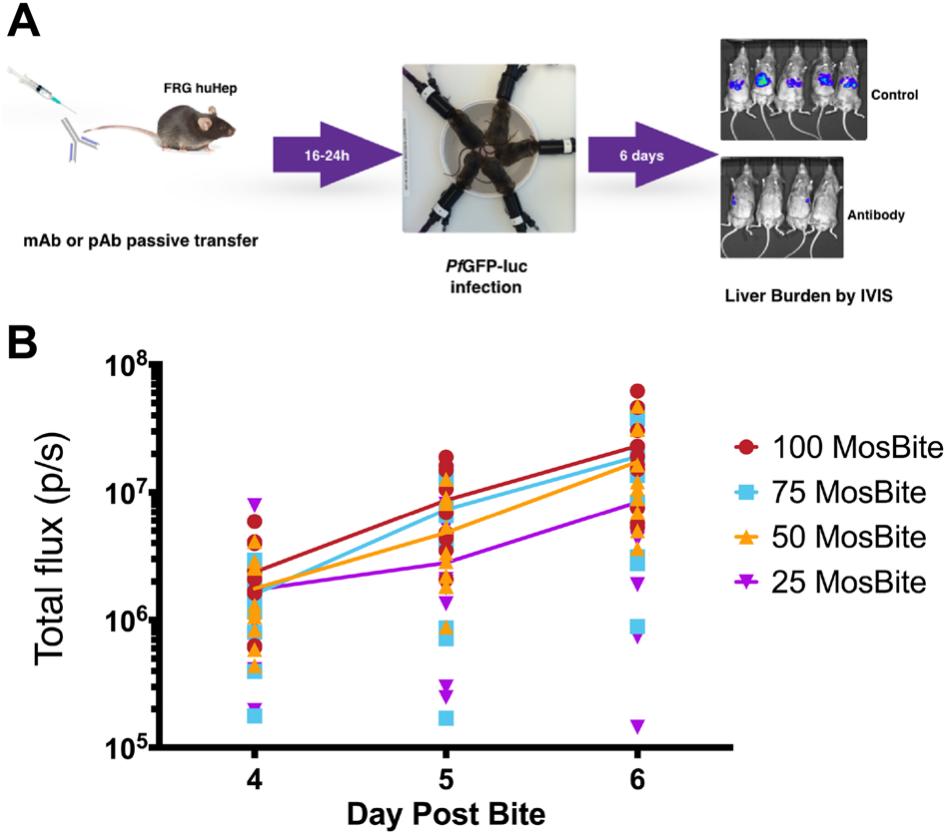
FRG huHep mice are susceptible to *P. falciparum* infection by mosquito bite and parasite liver stage burden can be measured noninvasively by bioluminescent imaging. **a** Overall schematic of passive transfer and mosquito bite infection of FRG huHep mice. Mice are administered antibody 16–24 h prior to infection with *Pf* GFP_luc-infected mosquitoes followed by subsequent assessment of liver stage burden by bioluminescence at 4–6 days post infection. **b** Mice were exposed to the bites of 25, 50, 75 or 100 mosquitoes and tracked for liver stage burden from days 4–6. Individual mice are shown as data points with the mean for each group shown by the color-corresponding connecting line

To determine antibody inhibitory function in vivo, we used the natural mosquito bite route of infection as opposed to intravenous (iv) injection of sporozoites, which has been used in the past but bypasses the skin stage and is less sensitive to antibody-mediated inhibition.^33,35^ However, mosquito bite infection is challenging to standardize, as it is difficult to control the precise sporozoite dose. Thus, we first asked if FRG huHep mouse infection with *Pf* parasites^36^ by mosquito bite yields a parasite stage liver burden of sufficient magnitude and consistency. We thus challenged FRG huHep mice with 25, 50, 75 or 100 *Pf* GFP_luc-infected mosquitoes each for 10 min. Liver stage burden was then quantified via bioluminescence on days 4–6 after infection (Fig. 1a). All mice had detectable liver infection on day 4 that increased on days 5 and 6 consistent with parasite liver stage growth over time (Fig. 1b). On day 6, all infections were statistically similar except the 25-bite group, which was significantly lower than both the 100 and 75 bite groups (*p* = 0.0021 and 0.0427, respectively, by two-way analysis of variance (ANOVA) for each day, Fig. 1b and Table 1). Given that 50 infected mosquito bites resulted in similar liver stage burden when compared to 75 and 100 mosquito bites (Table 1), we chose 50 bites as a standard dose for all further experiments. This dose proved to be consistent in terms of achieving a high magnitude of liver stage infection in all mice with a reasonable degree of variability that allowed us to detect antibody-mediated reductions in parasite liver infection in subsequent experiments (Supplementary Fig. S1).

**Table 1:** Mean liver burden and variation at day 6 post infection

Mosquito bites	*n*	Mean liver burden (p/s)	SD	SEM	Coefficient of variation
25	10	0.839 × 10^7^	0.686 × 10^7^	0.217 × 10^7^	81.7%
50	10	1.74 × 10^7^	1.451 × 10^7^	0.459 × 10^7^	83.4%
75	10	1.91 × 10^7^	1.623 × 10^7^	0.514 × 10^7^	85%
100	10	2.31 × 10^7^	1.852 × 10^7^	0.586 × 10^7^	80.2%

#### Noninvasive quantitation of *Pf* parasite burden in the liver following passive antibody transfer

**Fig. 2:**
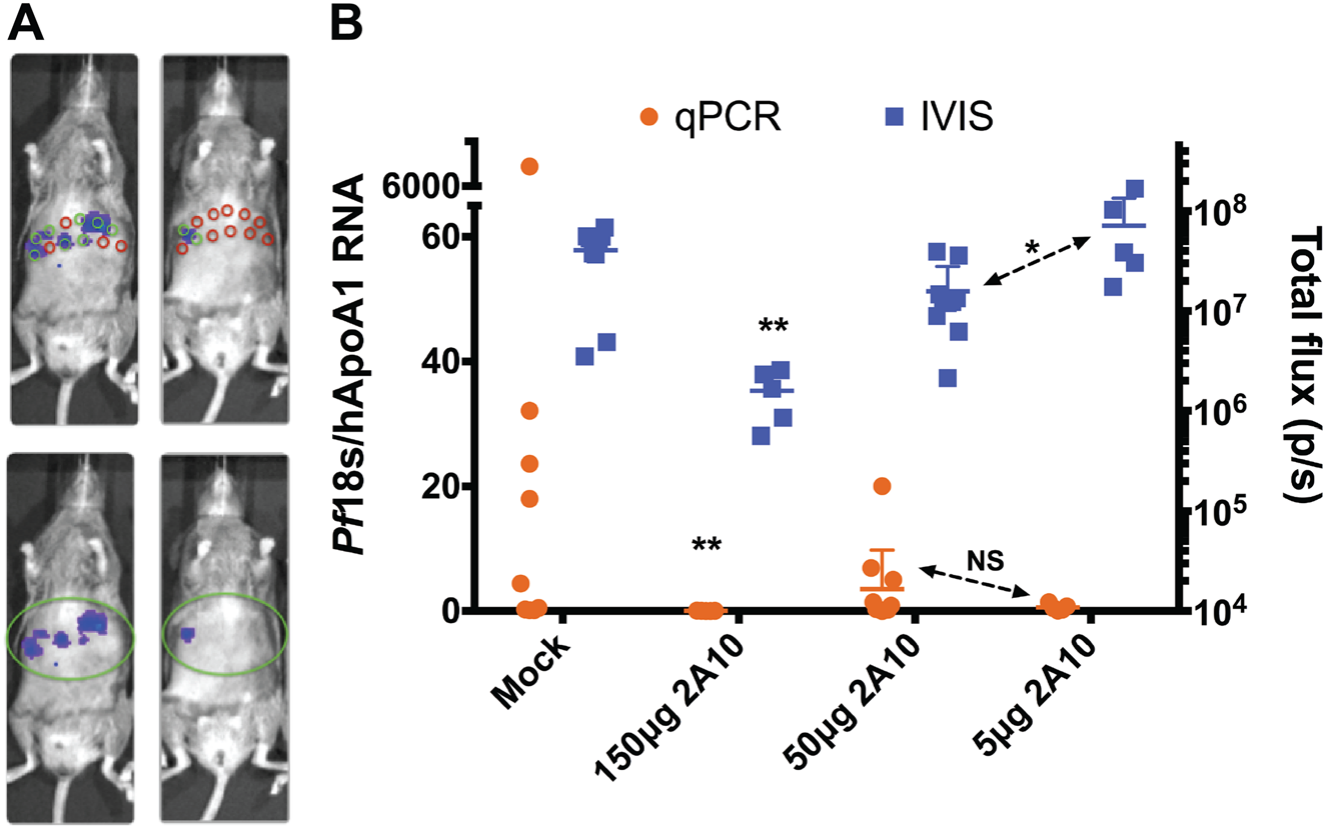
Bioluminescent imaging is as accurate as qRT-PCR for assessment of liver stage burden. **a** Schematic representing liver sampling with qRT-PCR (upper panels) and bioluminescence (lower panels). Sampling of livers with biopsy punch for qRT-PCR is indicated by 12 circles where green circles indicate samples in which parasite RNA would be detected and red those which would be negative in mice with high liver burden (left) and low liver burden (right). In contrast, assessment of liver burden by bioluminescence is shown in the bottom panels and indicates a representative region of interest that can capture the entire liver. **b** FRG huHep mice were administered indicated doses of anti-CSP mAb 2A10 or 150 μg of non-specific murine IgG (mock). The following day, mice were infected with 50 *Pf* GFP_luc mosquito bites and assessed for liver burden at day 6 by bioluminescent imaging. Immediately following imaging, mice were sacrificed and 12 samples from each liver were collected and combined for RNA isolation and quantitation of liver burden by qRT-PCR. Liver burden by both qRT-PCR (left *y*-axis) and bioluminescence (right *y*-axis) are shown with each data point representing the mean liver burden from the 12 liver sections from one mouse for qRT-PCR and the total flux for each mouse measured by bioluminescence

Bioluminescent imaging is a rapid and non-invasive method for assessing parasite liver stage burden. However, our initial experiments using mosquito bite infection showed variability within each experiment, which could preclude assessment of small changes in parasite burden such as those caused by moderate inhibition of infection with antibodies. Thus, we compared bioluminescent imaging and quantitative reverse transcriptase PCR (qRT-PCR) using parasite-specific probes to detect antibody-mediated reduction of liver infection. The latter method involves RNA extraction from infected liver and detection of parasite RNA by qRT-PCR.^6,31^ We compared these two methods by assessing the effect of passive transfer of 5, 50 and 150 μg of an anti-circumsporozoite protein (CSP) mAb on liver stage burden. A schematic example of sampling infected livers by both methods is shown in Fig. 2a. Six days after mosquito bite infection, liver stage burden was detectable in all mock-treated mice (mice receiving an equivalent dose of non-specific IgG) by both bioluminescent imaging and qRT-PCR (Fig. 2b) with considerable variability in both (coefficient of variance of 296 and 56% for qRT-PCR and bioluminescence, respectively). Both methods detected a significant reduction of liver stage burden following passive transfer of 150 μg of anti-CSP mAb with 3/5 mice having detectable liver burden by qRT-PCR and 5/5 by bioluminescent imaging (Fig. 2b). Whereas bioluminescent imaging could distinguish differences in liver stage parasite burden in mice given 5 and 50 μg of mAb, qRT-PCR could not, due to the large variability in signal (Fig. 2b). These results indicate that both bioluminescent imaging and this method of qRT-PCR detect relatively large differences in liver stage burden but that the former may be more useful in discriminating moderate reductions in liver stage burden.

### FRG huHep mouse challenge to assess functional activity of mAbs

**Fig. 3:**
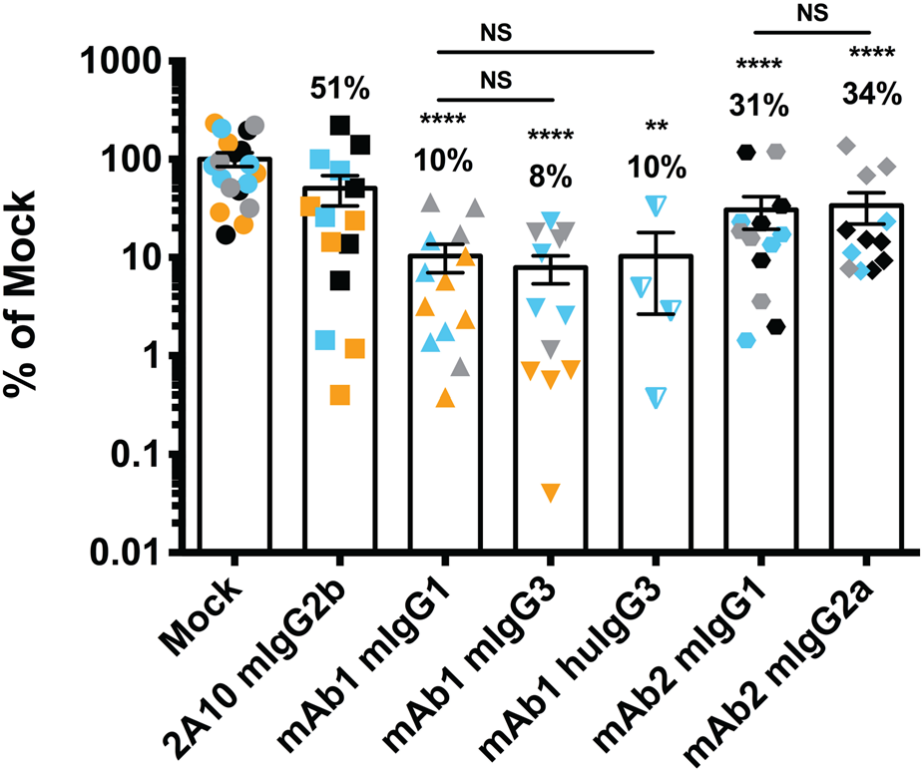
Monoclonal Abs of multiple Fc isotypes and species can be assessed in the FRG huHep/*Pf* GFP_luc challenge model. FRG huHep mice received 150 μg/mouse of non-specific IgG, mAb 2A10 or two novel anti-CSP mAb prior to mosquito bite infection and measurement of liver stage burden. Species and isotype are indicated for each mAb (e.g., mIgG1 for “murine IgG1” and “hu” for human). Data were collected over four independent experiments with 1–3 experiments/mAb and 4–5 mice/group/experiment. Each data point represents one mouse and mice within the same experiment are labeled in the same color. Bars indicate mean ± SEM with numbers above each bar indicating the mean % of mock for that group. Asterisks indicate statistical significance in one-way ANOVA comparing to mock-injected mice in the same independent experiments. Individual comparisons between mAb of the same specificity but different Fc isotypes/species are indicated with lines. ** is 0.01 > *p* > 0.001, **** is *p* < 0.0001

We next tested whether the FRG huHep/*Pf* GFP_luc challenge model could be used to measure differences in prevention of liver infection by distinct mAbs that target the same sporozoite antigen. To this end, we passively transferred three different mAbs of mouse origin that recognize the NANP repeat region of *Pf* CSP at 150 μg/mouse: mAb clone 2A10 (murine isotype IgG2b), mAb1 (murine isotype IgG1) and mAb2 (murine isotype IgG1). mAb 2A10 reduced liver infection to 51% of mock-treated mice while mAb1 and mAb2 reduced liver infection significantly to 10 and 31% of mock-treated mice, respectively (Fig. 3). To determine if the isotype of the mAb influenced antibody function, we also tested variants of mAb1 and mAb2 with isotypes known to better mediate Fc-dependent functions.^37^ Both mAb1-IgG3 and mAb2-IgG2a performed similarly to their IgG1isotype counterparts, indicating that antibody isotype was not a factor for in vivo performance in these experiments (Fig. 3). The results with these mAbs were consistent between replicate experiments (indicated by different colors in Fig. 3) despite differences in liver stage burden of mock groups (Supplementary Fig. S1). Importantly, the hierarchy in infection-inhibitory activity of each mAb, where mAb1 > mAb2> mAb 2A10, was not observed in an in vitro inhibition of sporozoite traversal and invasion (ISTI) assay where all mAbs performed similarly (Supplementary Fig. S2). This indicates that the assessment of antibody function in vitro may not fully reveal the inhibitory activity on sprozoite infection observed in vivo.

To increase the utility of the FRG huHep challenge model, we assessed whether mAbs of human origin can also be tested (i.e., derived from B cells from vaccinated volunteers). mAbs with human Fc regions set up a species mismatch between the antibody Fc region and the murine Fc receptor and this could diminish the contribution of Fc-dependent effector functions and obscure protection status.^38^ However, we observed that passive transfer of a version of mAb1 containing a human IgG3 (huIgG3) Fc region yielded similar results when compared to its murine counterparts (Fig. 3). Taken together, these data indicate that the FRG huHep/*Pf* GFP_luc challenge model discriminates distinct mAb blocking activities and enables functional assessment of mAbs of various Fc isotypes of both murine and human origin.

### Inhibitory activity of polyclonal IgG from whole sporozoite-immunized volunteers

We next assessed the inhibitory activity of human polyclonal antibodies collected after immunization of volunteers with live-attenuated *Pf* sporozoites.

**Fig. 4:**
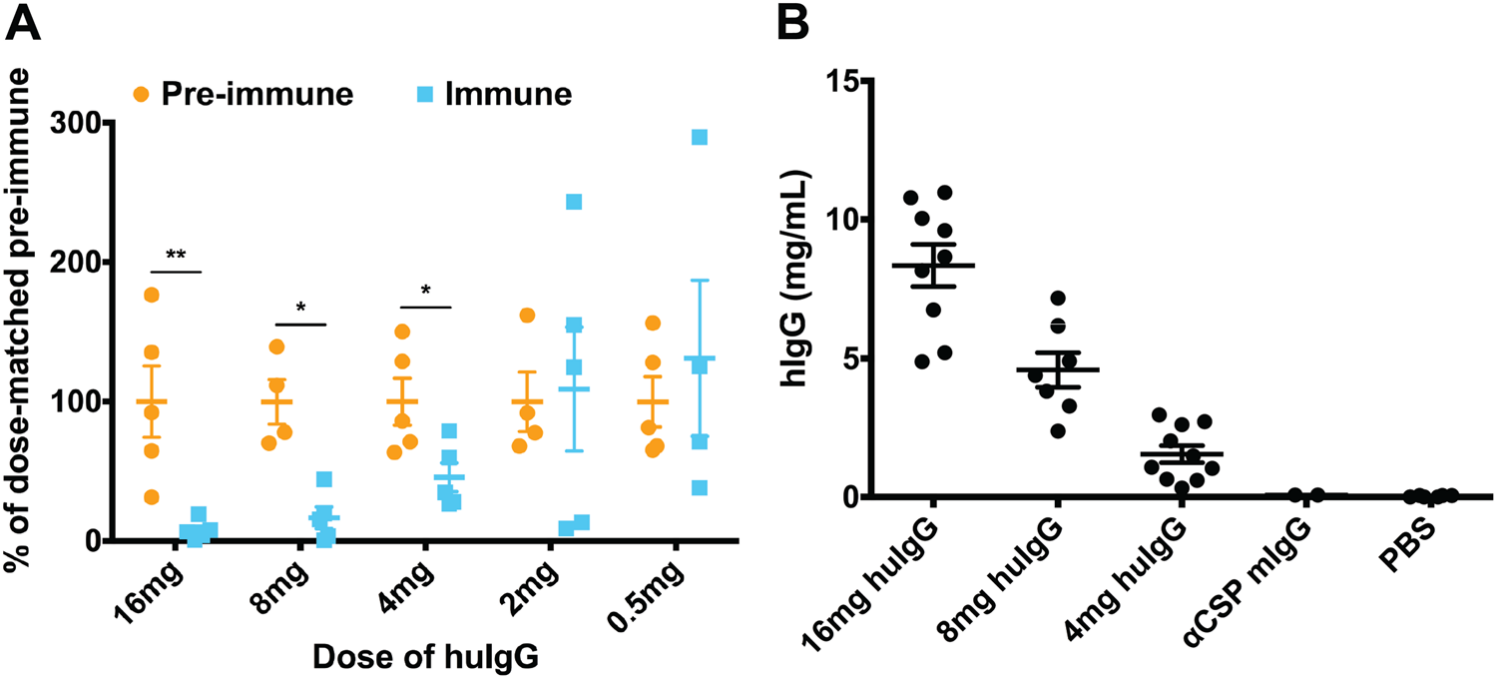
Human polyclonal IgG can be used over a wide dose range in the FRG huHep/*Pf* GFP_luc challenge model. FRG huHep mice (*n* = 4–5/group) received indicated doses of pooled pre-immune or immune polyclonal IgG at indicated doses. **a** Parasite liver stage burden was measured at day 6 post 50 mosquito bite challenge and the liver stage burden of each mouse receiving huIgG from immunized volunteers (Immune) was normalized to the average of the dose-matched huIgG from the pre-immunized volunteers (Pre-immune). Mean ± SEM is plotted with comparisons between pre-immune and immune liver stage burdens carried out by Mann–Whitney *U* test. Asterisks indicate a significant difference between means where * is *p* < 0.05 and ** is *p* ≤ 0.01. **b** Mice were bled immediately prior to challenge and serum was collected for measurement of circulating huIgG by ELISA. Concentration is depicted on the *y*-axis with mean ± SEM plotted for each group. Included are groups of mice that received a murine anti-CSP mAb or PBS (negative controls, *n* = 2 and 6, respectively)

Immune serum or plasma from malaria vaccine trials is often limited and in previous studies using passive transfer of huIgG, the dose of IgG was dictated by sample availability.^2,34^ Thus, to first characterize the range of huIgG inhibitory activity in the FRG huHep/*Pf* GFP_luc challenge model, polyclonal huIgG pooled from samples collected from whole sporozoite-immunized volunteers^2,34^ was passively transferred at 0.5, 2, 4, 8 and 16 mg/mouse. We detected significant reductions in parasite liver stage burden as compared to mice receiving equivalent doses of pre-immune huIgG at doses as low as 4 mg/mouse (Fig. 4a). The serum levels of huIgG in mice receiving these inhibitory doses at the time of challenge were an average of 1.5, 4.5 and 8.3 mg/mL for doses of 4, 8 and 16 mg/mouse, respectively (Fig. 4b). Together, these data indicate that a range of huIgG concentrations can be used to determine antibody functionality in the FRG huHep/*Pf* GFP_luc challenge model and that a dose of 16 mg/mouse can closely recapitulate the concentration of huIgG observed in human serum.^39^

**Fig. 5:**
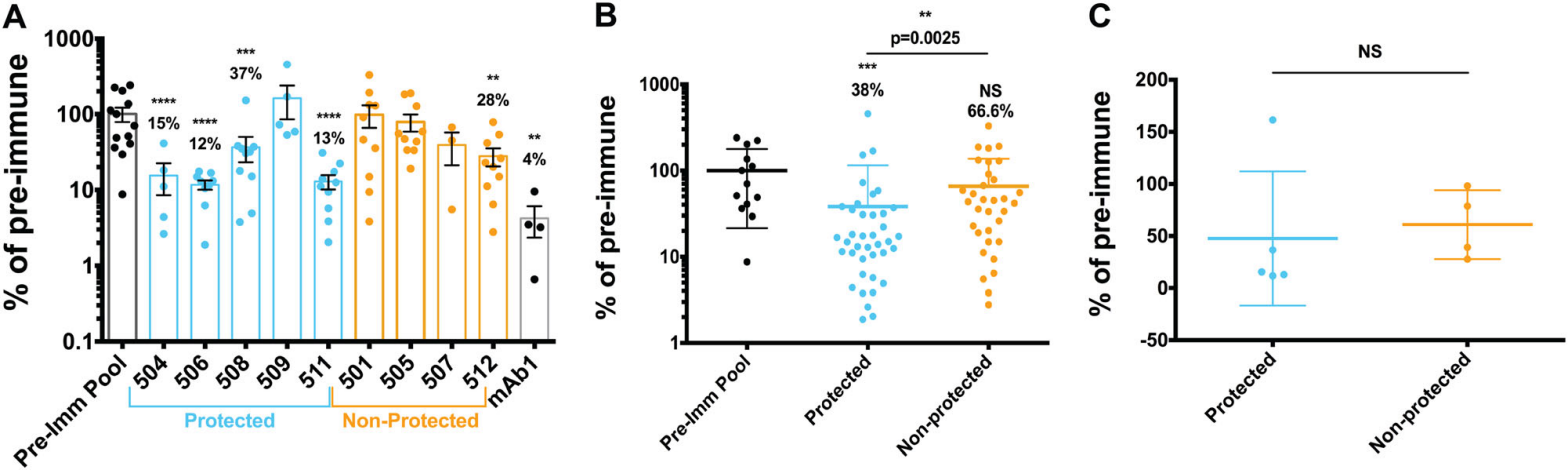
The FRG huHep/*Pf* GFP_luc challenge model can distinguish functional from non-functional antibodies from human volunteers. FRG huHep mice were administered 8 mg/mouse of IgG from volunteers 6 months after immunization with four doses of 2.7 × 10^5^ irradiated sporozoites (PfSPZ) and immediately prior to challenge by infectious mosquito bite. Mice were then infected by bite of 50 *Pf* GFP_luc-infected mosquitos and liver stage burden assessed at day 6 post infection. Data shown are a combination of two independent experiments as delineated in Figure S4 and expressed as a percent of pre-immune parasite liver burden (% of pre-immune). **a** Liver stage burdens of mice (*n* = 3–10/volunteer) receiving post-immunization IgG were normalized to mice receiving pooled pre-immune IgG (*n* = 13). Data points are shown with bars as mean ± SEM for each volunteer grouped by protection status. Asterisks indicate significant differences from the pre-immune group as measured by one-way ANOVA with Kruskal–Wallis post-test. **b** Data in **a** arranged such that each mouse is plotted as a single data point and grouped according to their treatment, pre-immune huIgG, immune huIgG from a CHMI protected volunteer or immune huIgG from a non-protected CHMI volunteer. Above each group is the mean % of pre-immune as well as asterisks indicating a significant difference from the mean of the pre-immune group as measured by one-way ANOVA with Kruskal-Wallis post-test. An additional Mann–Whitney test comparing the means of protected and non-protected is indicated by bar and resulting *p* value. **c** Data represented as one mean per volunteer with Mann-Whitney test used to determine if the mean % of mock are different. For all comparisons ** is *p* ≤ 0.01, *** is *p* ≤ 0.001 and **** is *p* ≤ 0.0001

We next used huIgG isolated from volunteers immunized with whole sporozoites and subsequently challenged by CHMI. The first set of huIgG was derived from volunteers intravenously immunized with four doses of 2.7 × 10^5^ purified, cryopreserved irradiated sporozoites (PfSPZ).^2^ CHMI 6 months after final immunization demonstrated protection in 6/11 volunteers.^2^ We obtained sufficient sample to transfer 8 mg of huIgG/FRG huHep mouse (*n* = 3–10 mice/volunteer) taken at the time of CHMI from five protected and four non-protected volunteers. Control FRG huHep mice received 8 mg/mouse of pooled pre-immune huIgG from all nine volunteers and all mice were challenged by 50 *Pf* GFP_luc-infected mosquito bites. Compared to mice receiving pooled pre-immune huIgG, the immune huIgG from 4/5 protected volunteers significantly reduced parasite liver infection while immune huIgG from only 1/4 non-protected volunteers demonstrated significant reduction of liver infection (Fig. 5a). When grouped together, mice receiving immune huIgG from protected volunteers showed significantly reduced parasite liver burden (38% of pre-immune) as compared to mice receiving huIgG from non-protected volunteers, which only had a non-significant (66.6% of pre-immune) reduction in liver stage burden (Fig. 5b). However, this correlation was not evident on an individual volunteer basis as the mean parasite liver burden of mice receiving huIgG from a single volunteer (i.e., 1 mean/volunteer) was not statistically lower for individuals in the protected group as compared to the non-protected group despite a lower numerical value (Fig. 5c). Thus, antibody inhibitory function in vivo was not a clear correlate of protection for volunteers in this study. Regardless, the observed trends in differences between protected versus non-protected huIgG were not detected using an in vitro ISTI assay where IgG from 3/3 non-protected volunteers tested significantly inhibited sporozoite invasion whereas IgG from only 3/5 protected volunteers showed significant inhibition of invasion (Supplementary Fig. S3A, B). Comparing in vitro inhibition of either cell traversal or invasion to FRG huHep/*Pf* GFP_luc challenge data also yielded no correlation between the two assays suggesting a fundamental difference between sporozoite infection in the in vitro assay and the in vivo assay (Supplementary Fig. S3C **)**. Finally, the in vivo data included a replicate with a subset of immune huIgG to assess the reproducibility. We found results to be consistent between experiments using different batches of *Pf* GFP_luc-infected infected mosquitoes (Supplementary Fig. S4).

**Fig. 6:**
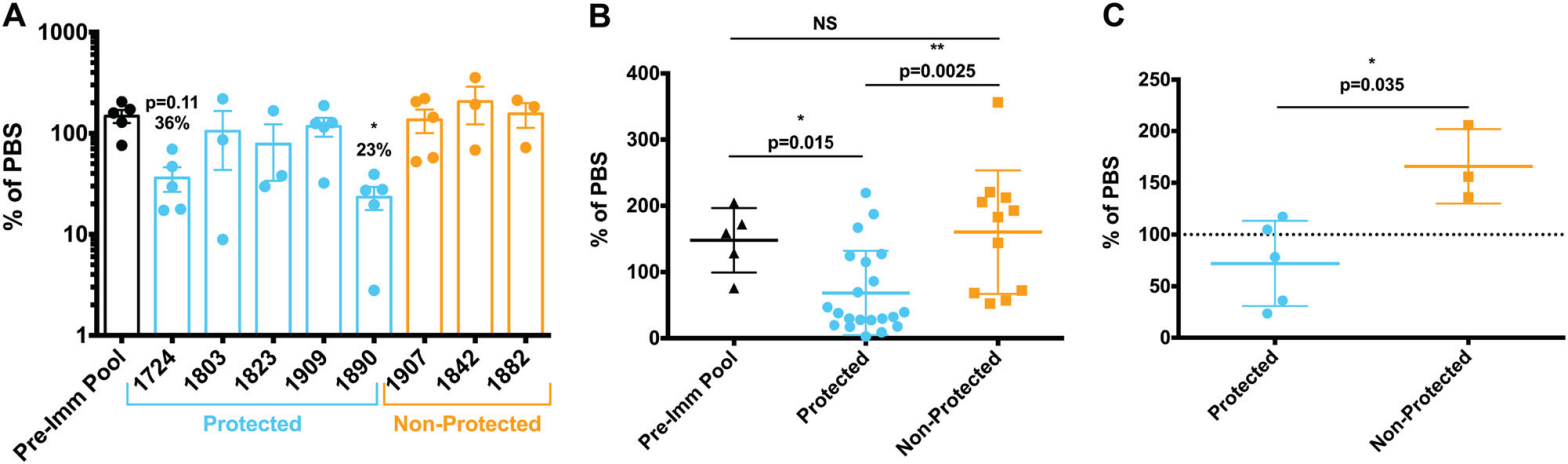
Antibody function in the FRG huHep/*Pf* GFP_luc challenge model can be used as a correlate of protection. FRG huHep were administered 5 mg/mouse of IgG collected from volunteers either before (“Pre-Imm”) or 2 weeks after three immunizations with 15 *Pf*-infected mosquito bites under chloroquine prophylaxis. Mice were infected by the bite of 50 *Pf* GFP_luc-infected mosquitos and liver burden assessed at day 6 post infection. **a** Parasite liver stage burdens of mice (*n* = 3–5/volunteer) receiving pooled pre-immunization or individual post-immunization huIgG were normalized to mice receiving an equivalent volume of PBS (% of PBS). Data points are shown with bars as mean ± SEM for each volunteer and grouped by protection status. Asterisks indicate significant differences from the pre-immune group as measured by one-way ANOVA with Kruskal–Wallis post-test. **b** Data in **a** but arranged such that each mouse is plotted as a single data point and grouped by treatment with pre-immune huIgG, immune huIgG from a protected volunteer or immune huIgG from a non-protected volunteer. Above each group is the mean % of PBS as well as asterisks indicating a significant difference from the mean of pre-immune as measured by one-way ANOVA with Kruskal–Wallis post-test. An additional Mann–Whitney test comparing the means of protected and non-protected is indicated by bar and resulting *p* value. **c** Data represented as one mean per volunteer with Mann–Whitney test used to determine if the mean % of PBS between mice receiving protected and non-protected huIgG are different. For all comparisons * is *p* < 0.05 and ** is *p* ≤ 0.01

We next tested a set of huIgG from volunteers immunized by the chloroquine prophylaxis with sporozoites (CPS) protocol.^40^ CPS administers fully infectious sporozoites by mosquito bite to volunteers that concurrently receive chloroquine, which leads to complete liver stage development, release of exo-erythrocytic merozoites and subsequent elimination of the first cycle of blood stage parasites. CPS has proven to be highly efficacious,^6,40,41^ and in this trial protected 5/8 volunteers from CHMI. Due to sample limitations, we only transferred 5 mg huIgG into each FRG huHep mouse (3–5 mice/volunteer). Controls received either pre-immune huIgG or only phosphate-buffered saline (PBS). There was no significant difference between pre-immune huIgG and PBS-treated mice (Supplementary Fig. S5A). When compared to mice receiving pre-immune huIgG, immune huIgG from 1/5 protected volunteers significantly reduced parasite liver stage burden with another non-significantly reducing liver stage burden to 36% of control (Fig. 6a). In contrast, immune huIgG from none of the three non-protected volunteers reduced parasite liver burden (Fig. 6a). Grouping of mice based on those that received immune huIgG from either protected or non-protected volunteers, as in Fig. 5b, revealed that the parasite liver stage burden of mice receiving “protected” immune huIgG was lower (68% of PBS) than mice receiving either pre-immune huIgG (148% of PBS) or “non-protected” immune huIgG (160% of PBS, Fig. 6b). Grouping the mean parasite liver stage burdens of mice receiving huIgG from a single volunteer (one mean/volunteer) as in Fig. 5c revealed a lower average parasite liver stage burden after passive transfer of “protected” huIgG (72% of PBS) as compared to “non-protected” huIgG (166%, Fig. 6c). Again, these in vivo results were not predicted by the in vitro ISTI assay (Supplementary Fig. S5B). Although limited by small sample sizes, these data together demonstrate that the FRG huHep/*Pf* GFP_luc challenge model can be used to distinguish functional from non-functional polyclonal antibodies derived from volunteers vaccinated with whole sporozoites. The trends we observed also suggest that this model could be used to establish correlates of protection based on the in vivo function of antibodies but will likely require analysis of vaccine trials with larger cohort sizes.

**Fig. 7:**
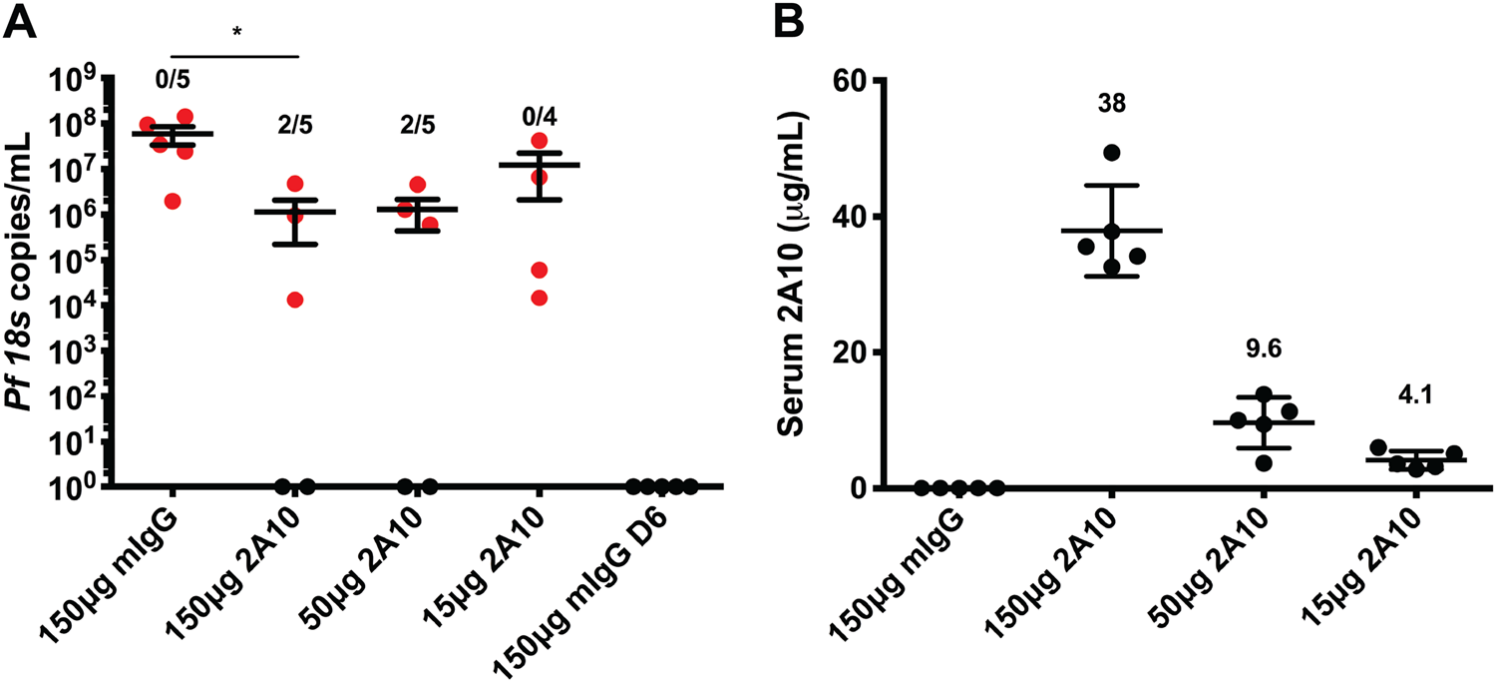
The FRG huHep/*Pf* challenge model can be used to assess sterile protection. FRG huHep mice (*n* = 4–5 mice/group) were administered indicated dose of mAb 2A10 or non-specific mIgG 1 day prior to challenge by bite of five *Pf*NF54-infected mosquitos. On day 6.5, mice were iv-injected with 400 μL of human red blood cells at 70% hematocrit. On day 7, peripheral blood was collected and used to assess presence of parasitemia by qRT-PCR. **a** Copies/mL of parasite 18S rRNA for each indicated dose of mAb 2A10 with negative mice plotted on the *x*-axis. Data points are individual mice with bars representing the mean ± SEM for each group. As a negative qRT-PCR control, mIgG-treated mice were bled prior to blood stage transition on day 6 (“150 μg mIgG D6”). Comparions between mIgG and 2A10-treated groups were carried out by one-way ANOVA and Kruskall–Wallis post-test. Significant differences are indicated by asterisk where *p* < 0.05. **b** Serum was collected immediately prior to challenge and circulating levels of mAb 2A10 were measured by ELISA. Each data point is one mouse with bars representing mean ± SEM shown for each group and numerical mean above each data set

The ultimate goal of malaria vaccination is sterile protection, i.e., the complete prevention of parasite egress from the liver and subsequent onset of blood stage infection. Measuring reduction of liver stage burden as shown here does not directly assess sterile protection. We addressed this by performing passive mAb transfer followed by challenge with five *Pf* NF54-infected mosquito bites (the number of infected mosquitoes used for CHMI) and used the transition to blood stage infection instead of parasite liver burden as an endpoint of protection. At this low challenge dose, reductions in liver infection cannot be studied by bioluminescent imaging as the luminescence derived from this smaller infection is at the limit of detection without antibody treatment. Mice in this experiment were given 150, 50 or 15μg/mouse of mAb 2A10 the day prior to challenge and injected with human red blood cells on day 6.5 to allow parasite transition into the blood. The following day (day 7), peripheral blood was taken and parasite density measured by *Plasmodium* 18S rRNA qRT-PCR. None of the mice treated with non-specific mIgG were protected as all mice had detectable blood stage infection. However, in mice that received 150 μg mAb/mouse, a dose which reduced liver burden by 49% after 50 mosquito bite challenge, 2/5 were sterilely protected from the 5 bite challenge. Reduction in dose to 50 μg/mouse also resulted in 2/5 mice protected while an even lower dose of 15μg failed to show any protection (Fig. 7a). Parasite densitites in infected mice that received 150 μg of mAb were 52-fold lower than mIgG-treated mice (*p* < 0.05). Unprotected mice in the 50 μg dose group and the 15μg group had non-significant reductions in parasite densitites by 45-fold and 4.8-fold, respectively. We also determined the circulating mAb levels at the time of challenge in these mice to be an average of 38, 9.6 and 4.1 μg/mL in the 150, 50 and 15 μg/mouse groups, respectively. Thus, in addition to determining reductions in parasite liver burden following passive transfer, FRG-huHep mice can be used to measure sterile protection using a natural mosquito bite challenge that is equivalent to CHMI using a qRT-PCR endpoint assay also used in CHMI studies.^34,42,43^

## Discussion

The malaria vaccine candidate RTS,S containing the imunodominant protein on the sporozoite surface, *Pf*CSP, has undergone extensive clinical testing. It has achieved modest protection in phase III trials that correlate with anti-CSP-antibody titers and with CD4^+^ T cell responses^3,4^—evidence that antibodies against sporozoite antigens can afford protection against malaria in humans. This is corroborated by decades of studies in rodent malaria models using both active immunization and passive transfer of mAbs that demonstrate infection-blocking activity.^9,33,35,44–47^ Furthermore, clinical trials using immunization with live-attenuated sporozoites elicit anti-sporozoite antibodies that inhibit sporozoite infection in vitro and in vivo, and in some cases these antibody titers and in vitro function correlate with protection.^1,2,6,48^ Targeting the liver stages of parasite infection with T cells also affords sterile protection in rodent malaria models.^49–51^ However, the clinical performance of T cell based malaria vaccines has been low.^52–54^

RTS,S partial efficacy provided the impetus to embark on the development of a second generation subunit vaccine that not only boosts the quality, magnitude and durability of anti-CSP antibodies, but also incorporates additional antibody targets capable of blocking *Pf* sporozoite infection. However, these efforts are encumbered by the lack of preclinical models for natural *Pf* sporozoite infection delivered by mosquito bite. Here, we optimized a regimen of *Pf* infection by mosquito bite in FRG huHep mice that is consistent and robustly measures antibody efficacy with adequate group sizes over sequential experiments. This challenge strategy also proved sensitive enough to noninvasively measure reductions in parasite liver stage burden following passive transfer of mAbs in a manner that was at least as sensitive as qRT-PCR of infected liver tissue. Our noninvasive bioluminescent readout was superior to a qRT-PCR method that avoids processing the entire liver, but increased sensitivity might be achieved if whole liver RNA preparation and qRT-PCR were used.^6,31,55^ In addition, bioluminescent imaging is less time and reagent intensive as demonstrated by the fact that the parasite liver stage burden of 60 mice can be assessed in only approximately 1.5 hours by a single experienced researcher.

The FRG huHep/*Pf* GFP_luc challenge model can thus be used to down-select mAbs based on their in vivo function prior to clinical advancement of either the mAb or targeted antigen. Indeed, we were able to discriminate low-performing anti-CSP mAbs such as clone 2A10 from those reducing parasite liver stage burden by up to 90%. Importantly, antibody efficacies were maintained after switching to different mouse IgG subclasses or human Fc. This is critical as mAb with human Fc (i.e., those isolated from vaccinated volunteers) could set up a mismatch between the human antibody and the FRG huHep mouse host. This mismatch could obscure Fc-dependent antibody function given the poor performance of human Fc regions in mice.^38^ While we did not see an impact of the Fc region on Ab performance, we only tested mAbs recognizing CSP, and previous reports have also shown that the Fc region of anti-CSP antibodies is dispensable.^44,56^ Thus testing of non-CSP specific antibodies could show different results in the FRG huHep mouse if Fc-dependent mechanisms exist for other antigen targets. One such antibody targeting the α-gal glycan is heavily Fc-dependent in a rodent malaria model.^57^ As similar antibodies become available for *Pf*, it will be important to study their efficacy in FRG huHep mice. Nevertheless, the FRG huHep/*Pf* GFP_luc challenge model is sensitive, flexible and medium throughput, allowing for the functional activity screening of both mAb and polyclonal IgG.

The in vivo efficacy of antibodies against single antigens, such as CSP, can also be tested in standard mice using transgenic rodent malaria parasites where the endogenous antigen of interest has been replaced by the *Pf* ortholog.^16–18^ However, creation of these parasites is complex and the *Pf* protein must fully complement the cognate rodent malaria parasite protein to yield a viable parasite. This becomes increasingly difficult if combinations of antibodies targeting multiple proteins is desired as parasites carrying all *Pf* proteins of interest must be made—requiring multiple rounds of transgenesis and increasing the likelihood of non-viable parasites. This approach is also not suitable for use with polyclonal antibodies with unknown specificities such as those derived from whole sporozoite immunizations which might elicit thousands of different antigen specificities.^58^ Polyclonal huIgG isolated from volunteers immunized with whole sporozoites have been tested for the ability to prevent liver stage infection in human liver-chimeric mice^2,6,34^ but none of these studies rigorously investigated the range of huIgG dose needed to determine protection nor have they investigated antibody function in vivo as a correlate of protection. Here, we determined that a wide range of polyclonal huIgG could be used to observe antibody function and a dose of 16 mg/mouse is needed to recapitulate levels of circulating huIgG found in human serum. Knowing these requirements will help guide sampling schedules in clinical trials where determining the function of vaccine-elicited antibodies in vivo is desired.

We also investigated polyclonal huIgG activity elicited after whole sporozoite immunization in vivo as a correlate of protection. Using huIgG from volunteers 6 months after immunization with the irradiated PfSPZ vaccine, we found a weak correlation between antibody function and protection of volunteers after CHMI. We observed a greater number of protected volunteers whose huIgG mediated significant inhibition of liver stage infection in FRG huHep mice, but the correlation between the ability of an individual’s huIgG to reduce liver stage burden and protection was not significant—largely due to a single volunteer whose huIgG resulted, surprisingly, in an increased liver stage burden. Thus, we could not establish a clear, predictive correlate of protection in this study. This could be due to limitations in antibody potency, the small sample size or the fact that antibodies may play a minor role in protection in this particular study. Immunization with irradiated sporozoites such as PfSPZ has been shown in animal models to be heavily dependent on liver-resident cytotoxic T cells,^59,60^ thus establishing a dominant correlate of protection using only antibody function might be unlikely. Still, our data add to the evidence that vaccination with irradiated sporozoites induces long-lasting antibody responses capable of reducing the number of sporozoites reaching the liver and is in agreement with previous research.^2^

In a further set of experiments using polyclonal huIgG from volunteers immunized by CPS, we also observed a weak correlation between in vivo huIgG function and protection from CHMI despite less robust antibody function in individual volunteers. Sample sizes were again small, but huIgG from protected volunteers reduced parasite liver stage burden to a greater degree than non-protected volunteers regardless of how the data were analyzed. While conclusions regarding correlates of protection in both of these trials should be tempered, it is clear that we were able to detect differences in antibody function between volunteers and between groups of distinct protection status. This suggests that similar studies using larger clinical cohort sizes could lead to the generation of statistically significant results and could be a critical tool for identifying correlates or mechanisms of protection in multi-antigen or whole parasite vaccination trials. Even if larger studies reveal no correlates of protection with humoral immunity, knowing which arms of the immune system mediate protection can help guide vaccine design or delivery to best target that immune compartment.

Importantly, we also show that FRG-huHep mice can be used to assess sterile protection when a highly sensitive *Plasmodium* 18 s rRNA qRT-PCR assay is used to detect blood stage infection after a lower, five mosquito bite challenge dose. This is the same challenge dose used in CHMI trials and is the same diagnostic endpoint assay used in such studies.^19,34,42,43^ All mock-treated mice in our sterile protection experiment became blood stage positive at day 7. Sterile protection is a binary readout with a limited dynamic range. However, while sterile protection was observed at the two highest doses of mAb 2A10 there was none in the lowest dose group, suggesting that this model is still sensitive to antibody dose and is reflective of overall efficacy. When coupled with the ability to modulate circulating mAb levels as shown here, it will be possible to determine the concentrations of antibody required to achieve sterile protection. This will be useful for screening and down-selection of antibodies against novel antigens or epitopes and the pre-CHMI determination of protective levels of antibodies that ought to be achieved by active immunization.

Finally, a key finding across all of our experiments was that the in vitro ISTI assay did not predict performance in the FRG huHep/*Pf* GFP_luc challenge model. The mAbs we tested all performed equally well in vitro yet exhibited large differences in reductions of parasite liver stage burden in vivo. In vitro results for clinical samples also did not predict any trends in inhibition and in fact over-estimated antibody function in non-protected volunteers. Thus, in vitro assays should be interpreted with caution and not used in isolation for down-selection of antibodies/targets, nor should they be used to rank antibody efficacy. It is perhaps not surprising that antibody function in vitro does not accurately predict function in vivo as the sporozoite uses unique mechanisms to travel through the dermis, into the circulation, across the liver sinusoidal barrier and finally into the three-dimensional architecture of the liver for liver stage development.^9,10,33,35,47^ All current in vitro assays employ infection of hepatocytes in monoculture which is not representative of the sporozoites’ complex journey to the liver or the architecture of the target organ.^26^

FRG huHep mice still suffer some limitations for use in antibody studies as they lack B, T, NK and NKT cells. This precludes the detection of antibody efficacy if it relies on interactions between Fc regions and the Fc-receptors (FcR) on these cell types, although to our knowledge, there is no demonstration of antibody function against sporozoites being dependent on interacting with these cell types. Nevertheless, FRG huHep mice do harbor antigen-presenting cells such as dendritic cells and macrophages which could phagocytose antibody-bound sporozoites.^61^ Polymorphonuclear cells are also present in these mice^62^ and could augment antibody-mediated prevention of liver infection.^57^ However, all bone marrow-derived cells will contain mouse FcR that bind poorly to human Fc regions and may limit the function of transferred human mAb or huIgG.^38^ While the role of complement in protection against sporozoite infection is unclear,^44,56,57^ it is also unclear if these mice can fix complement via the classical/antibody-mediated pathway with either mouse or human antibodies. FRG huHep mice do make human C3 (data not shown), but it remains to be seen if this is functional in the classical complement pathway.

Despite their limitations, our data show that FRG huHep mice are a highly relevant laboratory model to study the role of inhibitory antibodies against natural *Pf* infection. mAbs against novel epitopes of existing antigen candidates or completely novel candidates can be screened in FRG huHep mice and will thereby help determine those that function best in vivo. The data can then help guide rational structure-based antigen design as well as down-select antigen candidates to move into CHMI trials using either passive immunization with mAbs or active immunization with the target antigen. Human liver-chimeric mice are also suitable models to test the in vivo function of polyclonal antibodies with diverse or unknown specificities such as those derived from vaccination with complex immunogens, including whole sporozoites. Testing of these antibodies in vivo will help identify correlates or contributing mechanisms of protection that cannot be discerned in vitro. Thus, the model described here can further enable rational optimization of protective malaria vaccine candidates and as such will form a bridge to clinical malaria vaccine development efforts.

## Materials and methods

### FRG huHep mouse challenges

Human hepatocyte donor-matched FRG huHep mice (both male and female, >4 months of age) were purchased from Yecuris, Inc. Repopulation of human hepatocytes was confirmed by serum albumin levels, and only animals with serum albumin levels >3 mg/mL were used. For passive transfer, mice were intravenously or intraperitoneally injected with indicated dose of anti-*Pf* CSP monoclonal antibody or huIgG 16–24 h prior to challenge. mAbs were kindly provided by PATH Malaria Vaccine Initiative where mAb1 is clone 3C1 and mAb2 is clone 2H8. For mosquito bite challenge, *Anopheles* mosquitos infected with *Pf* expressing GFP-luciferase or wild type NF54 were generated as previously described.^36^ Mosquito infection was quantified by midgut dissection at day 7–10 post-blood meal and were used only if >50% of mosquitos contained an average of >10 oocysts/midgut. All qualifying mosquitos were then pooled and re-distributed into cages with ~50 mosquitos/mouse with up to 250 mosquitos. For five-mosquito bite challenge, the number of mosquitos/mouse was adjusted to reflect infection prevalence (e.g., if 90% of mosquitos were infected, a total of 28 mosquitos were added to a cage to infect five mice). Mice, in groups of up to five, were then anesthetized with isoflurane and placed on a mesh screen covering the container of mosquitos while under isoflurane anesthesia via nose cone. Mosquitos were then allowed to feed for 10 min with lifting of mice every minute to encourage probing and injection of sporozoites rather than blood feeding. After 10 min mice were returned to normal activity. At day 6 post infection (peak of liver stage burden), mice were imaged for liver stage burden using bioluminescence and IVIS imaging as previously described.^33,63^ Briefly, mice were intraperitoneally injected with 100 μL of Rediject D-luciferin (Perkin Elmer) and imaged after 5 min for a five-minute exposure. Liver stage burden was assessed by placing an identical region of interest around the liver of each mouse and measuring total flux in pixels/second (p/s). Liver stage burden of all mice was normalized by setting the mean of the negative control group that received non-specific, species-matched IgG or pre-immune huIgG to 100% within each bite experiment. Liver stage burden was alternatively assessed in a subset of experiments by *Plasmodium* 18S rRNA RT-PCR normalized to hApoA1 mRNA, as previously reported.^6,64^

For assessment of sterile protection, mice were iv-injected with 400 μL of human red blood cells at 70% hematocrit in RPMI 6.5 days post challenge. On day 7, mice were bled via the retroorbital plexus and exactly 50 μL of blood was placed into 1 mL of Nuclisens EasyMag buffer (bioMérieux) and stored at −80 °C until extraction. Total RNA was extracted using an EasyMag instrument (bioMérieux) as described.^43^ *Plasmodium* 18S rRNA qRT-PCR was performed as described^65^ although newly described pan-*Plasmodium* 18S rRNA-specific reagents were used herein.^66^ Mice were considered positive if qRT-PCR was above the undetectable qRT-PCR signal obtained on day 6 post challenge.

### ELISA for serum mAb concentration

Mice were bled via retroorbital plexus immediately prior to challenge and blood allowed to clot in BD serum separator tubes for 2 h. Serum was separated by centrifugation at 14,000×*g* for 2 min. For ELISA, Costar EasyWash (Corning) were coated with 2 μg/mL in coating buffer as previously described.^34^ Plates were then blocked with dilution/blocking buffer (0.05% Tween-20, 6% bovine serum albumin in PBS) for 1 h at room temperature. After washing, 50 μL of serum samples were applied in duplicate at 1:160 and 1:320 dilutions in dilution/blocking buffer. A standard curve was generated using eight two-fold dilutions of 2A10 starting at 625 ng/mL in dilution/blocking buffer and 50 μL applied in duplicate with samples. Standards and serum samples were incubated at room temperature for 2 h. After washing, HRP-conjugated anti-mouse IgG was applied at a 1:5000 dilution for 1 h at room temperature. Plates were developed with SureBlue™ TMB reagent for 4 min and stopped with SureBlue™ Stop reagent before reading absorbance at 450 nm. Serum concentrations were interpolated using a 4-parameter non-linear regression of the standard curve.

### Ethics statement

All animal procedures were conducted in accordance with and approved by the Center for Infectious Disease Research Institutional Animal Care and Use Committee (IACUC) under protocol SK-16. The Seattle Biomed IACUC adheres to the NIH Office of Laboratory Animal Welfare standards (OLAW welfare assurance # A3640-01).

### Preparation of monoclonal and polyclonal antibodies

Monoclonal antibody 1 (mAb 1, clone 3C1) was prepared as previously described.^2^ Monoclonal antibody 2 (mAb 2, clone 2H8) was generated under a Gennova-PATH Malaria Vaccine Initiative collaborative program by immunization of mice against full length *Pf*CSP produced by Gennova. Hybridoma clone 2A10 specific for *Pf* CSP was obtained from MR4 and ProMab Biotechnologies, Inc. performed antibody production and purification. For IgG isolated from human serum, samples were prepared as recently published.^2^ Briefly, serum was used for extraction of IgG using protein G columns (GE Healthcare Life Sciences) following manufacturer’s protocol and concentrated using Amicon Ultra-15 centrifugal units (EMD Millipore) to 20–25 mg/mL in PBS.

### In vitro inhibition of sporozoite invasion and traversal assay (ISTI)

Antibodies and IgG were tested at indicated concentrations in ISTI following previously published methods.^67,68^ Briefly, freshly dissected *Pf* sporozoites were incubated with antibodies at indicated concentrations in DMEM media containing 10% heat-inactivated FBS supplemented with glutamine, penicillin/streptomycin, Fungizone and FITC-dextran for 15 min a 37 °C. Sporozoites and media were then added to HC04 cells (ATCC, Inc.) plated 1 day prior at 10^5^ cells/well in a 96-well plate at an MOI of 0.3 (sporozoites:HC04 cells). Plates were then spun at 500×*g* for 3 min and incubated for 90 min at 37 °C. Cells were then washed, trypsinized and transferred to a new 96-well plate where they were fixed and permeabilized using BD cytofix/cytoperm (BD Biosciences). Cells were then stained with anti-CSP mAb conjugated to AlexaFluor-647 and analyzed by flow cytometry using a BD LSRII and FloJo analysis software. Cells were considered “invaded” if they were CSP-positive and “traversed” if they had taken up FITC-dextran due to cell membrane wounding. Antibody-treated wells were normalized to either species-matched non-specific IgG for mAbs and to pooled pre-immune IgG at equivalent concentrations. Anti-CSP mAb 2A10 was included at 10 μg/mL in each assay as a positive control for inhibition.

### Statistical analysis

Relevant statistical tests are indicated in figure legends and were conducted using GraphPad Prism 6.0 for Mac. Significant results are indicated in figures with relevant *p* values indicated by “*” in each figure legend. Comparisons not indicated in figures were non-significant with *p* > 0.05.

### Data availability

The data that support the findings of this study are available from the corresponding author upon reasonable request.

## Electronic supplementary material

Supplementary Figure 1

Supplementary Figure 2

Supplementary Figure 3

Supplementary Figure 4

Supplementary Figure 5

Supplementary Figure Legends
