# Supplementary figures and images for: Humoral protection against mosquito bite-transmitted Plasmodium falciparum infection in humanized mice
Source: NPJ Vaccines. 2017 Oct 9;2:27. doi: 10.1038/s41541-017-0028-2 (PMC5634440; doi:10.1038/s41541-017-0028-2)

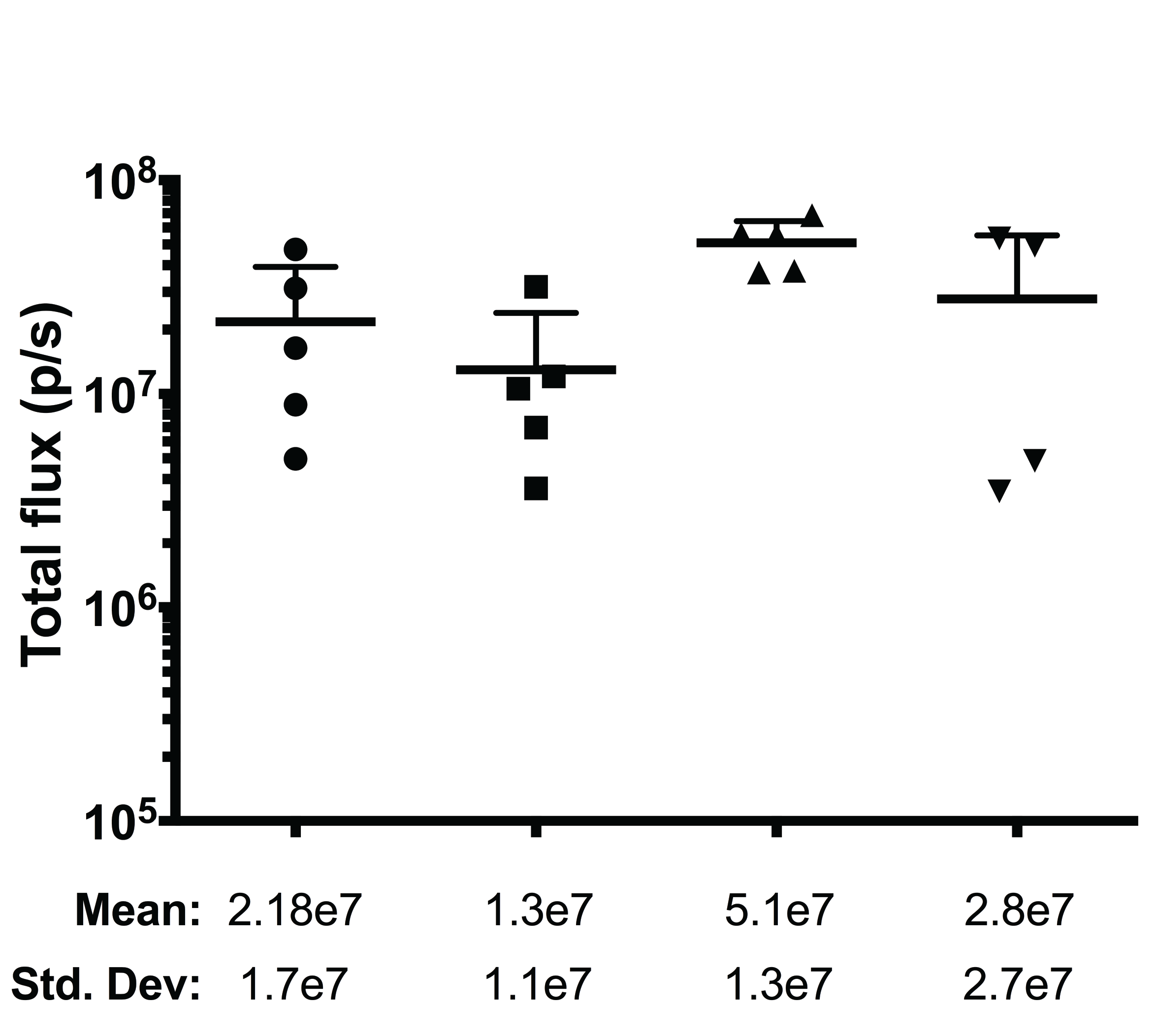

Supplement: Supplementary file 1 — Supplementary Figure 1 [file 41541_2017_28_MOESM1_ESM.png]

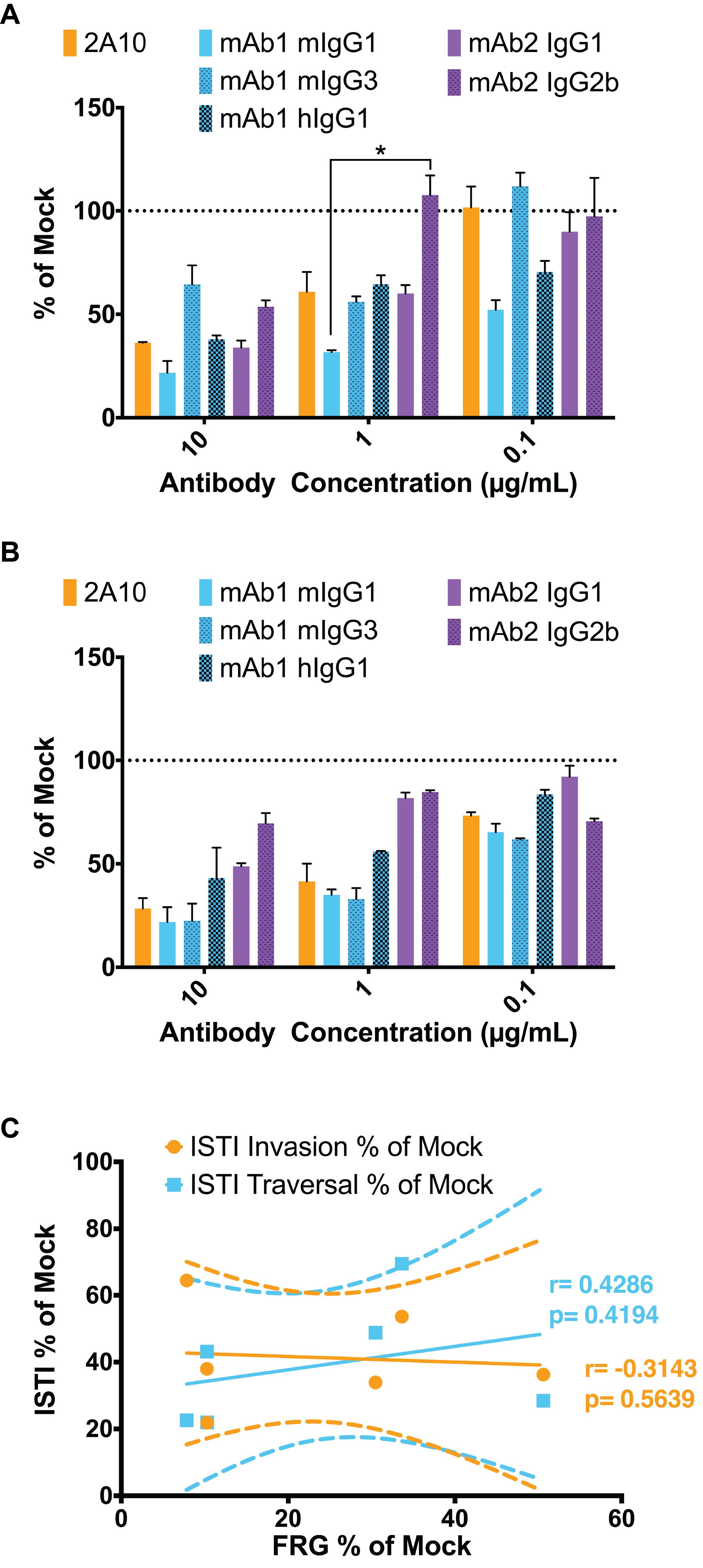

Supplement: Supplementary file 2 — Supplementary Figure 2 [file 41541_2017_28_MOESM2_ESM.png]

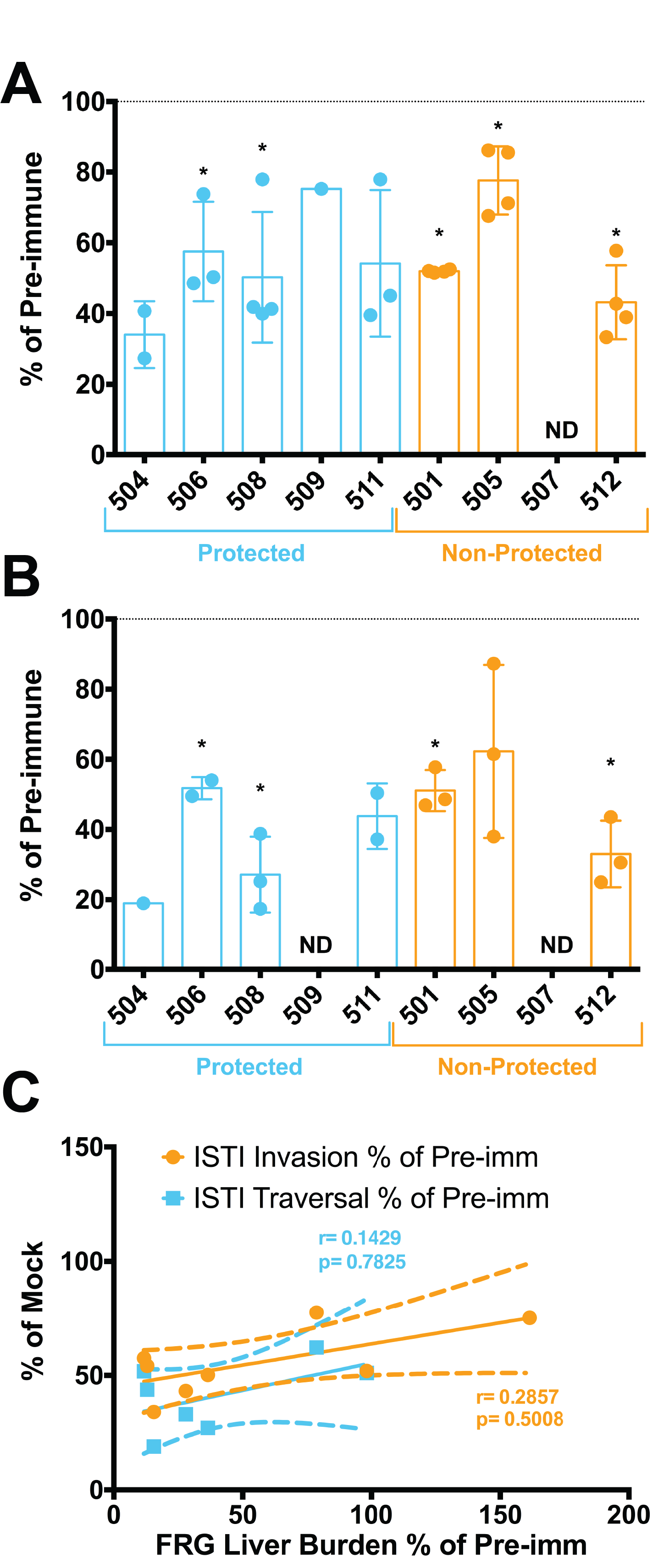

Supplement: Supplementary file 3 — Supplementary Figure 3 [file 41541_2017_28_MOESM3_ESM.png]

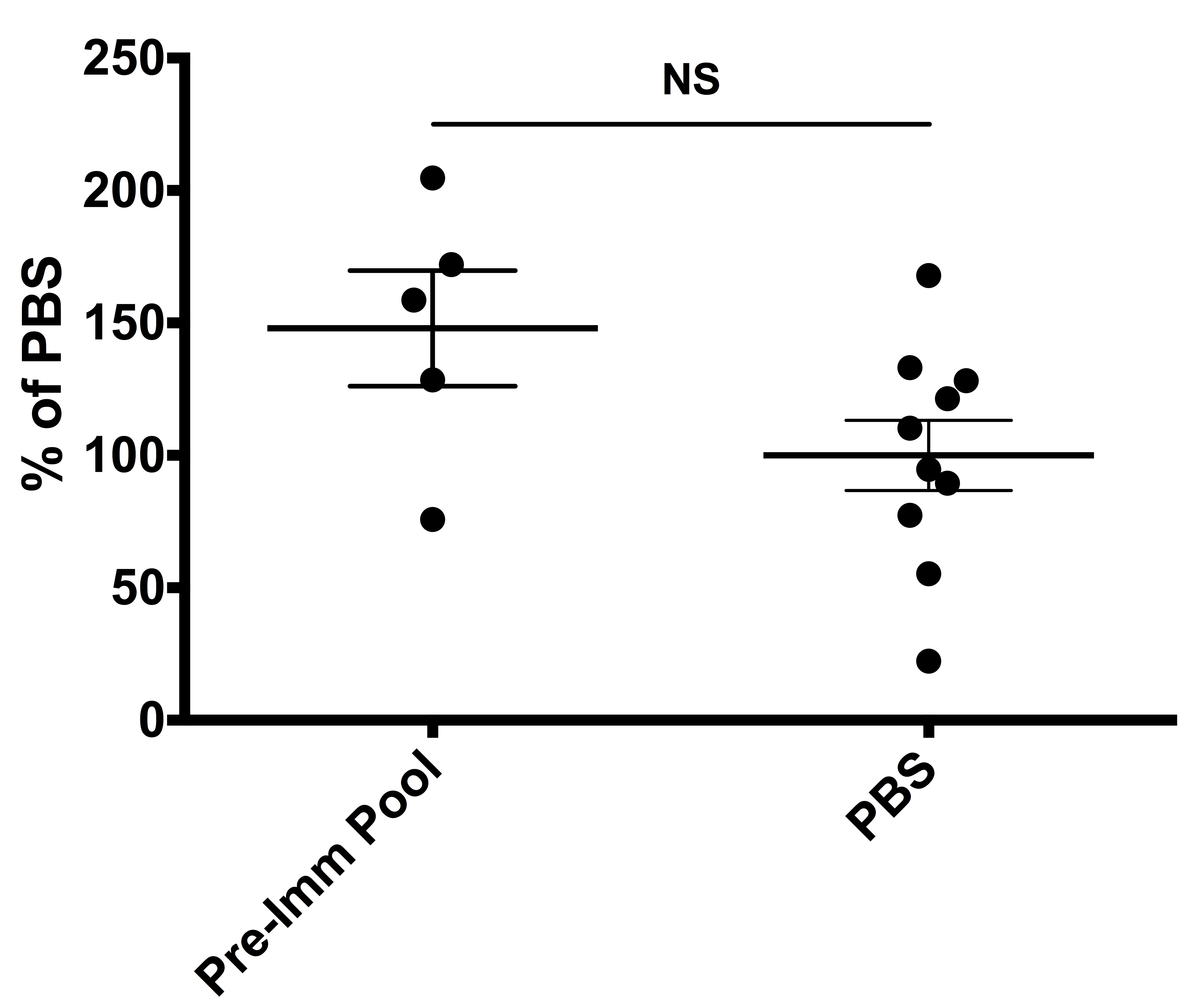

Supplement: Supplementary file 4 — Supplementary Figure 4 [file 41541_2017_28_MOESM4_ESM.tif]

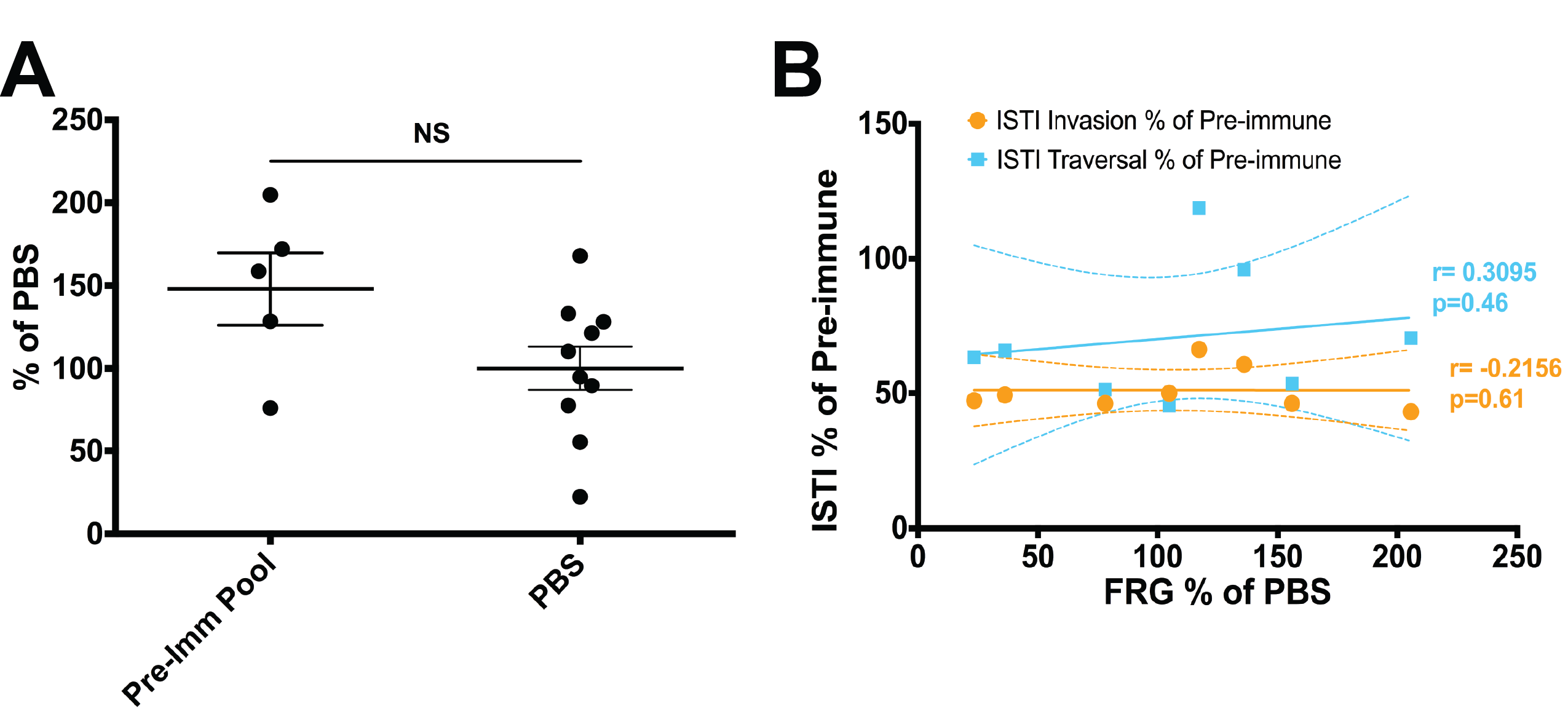

Supplement: Supplementary file 5 — Supplementary Figure 5 [file 41541_2017_28_MOESM5_ESM.png]
