# Supplementary material for: Humoral protection against mosquito bite-transmitted Plasmodium falciparum infection in humanized mice
Source: NPJ Vaccines. 2017 Oct 9;2:27. doi: 10.1038/s41541-017-0028-2 (PMC5634440; doi:10.1038/s41541-017-0028-2)
Supplement: Supplementary file 6 — Supplementary Figure Legends [file 41541_2017_28_MOESM6_ESM.docx]

**Supplementary Materials**

**Fig. S1.** **50-mosquito bite infection is consistent and robust**. Comparison of liver stage burdens across mock-treated groups from 4 independent experiments. Parasite liver stage burdens are assessed as total flux in pixels/second (p/s). Each data point is 1 mouse (n=4-5/experiment) and bars represent the mean with standard deviation. Below the graph are the numerical means along with standard deviation for each.

**Fig. S2.** ***In vitro* inhibition of sporozoite traversal and invasion assay does not predict mAb function *in vivo***. Monoclonal antibodies were tested in an ISTI at 0.1, 1 and 10 μg/mL. Sporozoite invasion (in **A**) and traversal (in **B**) were measured and normalized to wells containing equal concentrations of non-specific IgG. Bars represent mean of 2-3 independent experiments per mAb. To compare differences between mAbs, one-way ANOVA was used to compare the means within each concentration group and significant differences noted with an “*” where “*” is p<0.05. In **C**, values for sporozoite traversal and invasion at 10μg/mL are plotted against % of mock liver stage burdens as tested in FRG huHep passive transfer. Test for correlation was carried out by Spearman test with associated r and p values for each measure shown in color-matched text.

**Fig. S3. *In vitro* inhibition of sporozoite traversal and invasion assay does not predict function of IgG from human sera *in vivo*.** Polyclonal IgG isolated from volunteers immunized with PfSPZ (as in **Fig.** 5) was tested *in vitro* in an ISTI based on sample availability at 1 mg/mL. Sporozoite invasion (in **A**) and traversal (in **B**) were measured and normalized to pooled pre-immunization IgG. Data points are the result of individual, independent repeat experiments (n=2-4/sample, with n=1 for 504 traversal) with bars representing the means ± SD. A one-sample t-test to determine if means were significantly different than 100 % of mock was carried out and significant reductions are indicated by an “*” where “*” is p<0.05. In **C**, values for ISTI are plotted against % of mock liver stage burdens as tested in FRG huHep passive transfer. Test for correlation was carried out by Spearman test with associated r and p values for each measure shown in color-matched text.

**Fig. S4. Detection of inhibition of parasite liver stage burden in FRG huHep mice using polyclonal IgG is highly reproducible.** Passive transfer and infection of FRG huHep mice was carried out exactly as in **Figure 5** with pre-immune IgG and six blinded immune IgG samples from the three protected and three non-protected individuals tested in **Figure 5**. Data from this second experiment are represented by purple stars overlaid with their corresponding samples from Experiment 1.

**Fig. S5 Comparison of PBS vs. pre-immune-treated FRG huHep mice and lack of correlation between *in vitro* and *in vivo* assays using immune IgG from human sera.** Comparison of parasite liver burden in mice given 5 mg/mouse of pre-immune IgG or an equivalent volume of PBS prior to mosquito bite challenge as performed in **Figure 6** with Mann-Whitney test for differences in mean (in **A**). Inhibition of parasite invasion and traversal by polyclonal IgG used in **Figure 6** was tested in an ISTI at 1mg/mL in a single experiment (in **B**). Correlation of invasion or traversal with parasite liver burden was tested using Spearman test with associated r and p values for each measure shown in color-matched text.
